# Supplementary material for: Novel Anti-Cancer Products Targeting AMPK: Natural Herbal Medicine against Breast Cancer
Source: Molecules. 2023 Jan 11;28(2):740. doi: 10.3390/molecules28020740 (PMC9863744; doi:10.3390/molecules28020740)
Supplement: Supplementary file 1 [file molecules-28-00740-s001.zip › molecules-2036126-supplementary.pdf]

Supplementary information

Novel Anti-cancer Products Targeting AMPK: Natural Herbal Medicine Against Breast Cancer

Bo Peng †, Si-Yuan Zhang †, Ka Iong Chan, Zhang-Feng Zhong *, Yi-Tao Wang *

Macao Centre for Research and Development in Chinese Medicine, State Key Laboratory of Quality Research in Chinese Medicine, Institute of Chinese Medical Sciences, University of Macau, Macao SAR 999078, China

* Correspondence: zfzhong@um.edu.mo and ytwang@um.edu.mo

† These authors contributed equally to this work.

Table S1. Gene symbol and description of the genes mentioned in the manuscript [1–8].

Gene symbol	Gene ID	Official full name	Gene type
BMI1	648	BMI1 proto-oncogene, polycomb ring finger	protein coding
BRCA1	672	BRCA1 DNA repair associated	protein coding
MEDAG	84935	mesenteric estrogen dependent adipogenesis	protein coding
NANOG	79923	Nanog homeobox	protein coding
PFKFB3	5209	6-phosphofructo-2-kinase/fructose-2,6-biphosphatase 3	protein coding
PIK3CA	5290	phosphatidylinositol-4,5-bisphosphate 3-kinase catalytic subunit alpha	protein coding
PRKAA2	5563	protein kinase AMP-activated catalytic subunit alpha 2	protein coding
SOX2	6657	SRY-box transcription factor 2	protein coding

References

1. National Library of Medicine. BMI1 BMI1 proto-oncogene, polycomb ring finger [*Homo sapiens* (human)]. Available online: https://www.ncbi.nlm.nih.gov/gene/648 (accessed on 17 October 2022)
2. National Library of Medicine. BRCA1 BRCA1 DNA repair associated [*Homo sapiens* (human)]. Available online: https://www.ncbi.nlm.nih.gov/gene/672 (accessed on 17 October 2022)
3. National Library of Medicine. MEDAG mesenteric estrogen dependent adipogenesis [*Homo sapiens* (human)]. Available online: https://www.ncbi.nlm.nih.gov/gene/84935 (accessed on 17 October 2022)
4. National Library of Medicine. NANOG Nanog homeobox [*Homo sapiens* (human)]. Available online: https://www.ncbi.nlm.nih.gov/gene/79923 (accessed on 17 October 2022)
5. National Library of Medicine. PFKFB3 6-phosphofructo-2-kinase/fructose-2,6-biphosphatase 3 [*Homo sapiens* (human)]. Available online: https://www.ncbi.nlm.nih.gov/gene/5209 (accessed on 17 October 2022)
6. National Library of Medicine. PIK3CA phosphatidylinositol-4,5-bisphosphate 3-kinase catalytic subunit alpha [*Homo sapiens* (human)]. Available online: https://www.ncbi.nlm.nih.gov/gene/5290 (accessed on 17 October 2022)
7. National Library of Medicine. PRKAA2 protein kinase AMP-activated catalytic subunit alpha 2 [*Homo sapiens* (human)]. Available online: https://www.ncbi.nlm.nih.gov/gene/5563 (accessed on 17 October 2022)
8. National Library of Medicine. SOX2 SRY-box transcription factor 2 [*Homo sapiens* (human)]. Available online: https://www.ncbi.nlm.nih.gov/gene/6657 (accessed on 17 October 2022)
